# RAFTS^3^G: an efficient and versatile clustering software to analyses in large protein datasets

**DOI:** 10.1186/s12859-019-2973-4

**Published:** 2019-07-15

**Authors:** Bruno Thiago de Lima Nichio, Aryel Marlus Repula de Oliveira, Camilla Reginatto de Pierri, Leticia Graziela Costa Santos, Alexandre Quadros Lejambre, Ricardo Assunção Vialle, Nilson Antônio da Rocha Coimbra, Dieval Guizelini, Jeroniza Nunes Marchaukoski, Fabio de Oliveira Pedrosa, Roberto Tadeu Raittz

**Affiliations:** 1 Laboratory of Bioinformatics, Professional and Technical Education Sector from the Federal University of Paraná, Curitiba, PR Brazil; 2 Department of Biochemistry, Biological Sciences Sector – Federal University of Paraná (UFPR), Curitiba, PR Brazil

## Abstract

**Background:**

Clustering methods are essential to partitioning biological samples being useful to minimize the information complexity in large datasets. Tools in this context usually generates data with greed algorithms that solves some Data Mining difficulties which can degrade biological relevant information during the clustering process. The lack of standardization of metrics and consistent bases also raises questions about the clustering efficiency of some methods. Benchmarks are needed to explore the full potential of clustering methods - in which alignment-free methods stand out - and the good choice of dataset makes it essentials.

**Results:**

Here we present a new approach to Data Mining in large protein sequences datasets, the *Rapid Alignment Free Tool for Sequences Similarity Search to Groups* (RAFTS^3^G), a method to clustering aiming of losing less biological information in the processes of generation groups. The strategy developed in our algorithm is optimized to be more astringent which reflects increase in accuracy and sensitivity in the generation of clusters in a wide range of similarity. RAFTS^3^G is the better choice compared to three main methods when the user wants more reliable result even ignoring the ideal threshold to clustering.

**Conclusion:**

In general, RAFTS^3^G is able to group up to millions of biological sequences into large datasets, which is a remarkable option of efficiency in clustering. RAFTS^3^G compared to other “standard-gold” methods in the clustering of large biological data maintains the balance between the reduction of biological information redundancy and the creation of consistent groups. We bring the binary search concept applied to grouped sequences which shows maintaining sensitivity/accuracy relation and up to minimize the time of data generated with RAFTS^3^G process.

**Electronic supplementary material:**

The online version of this article (10.1186/s12859-019-2973-4) contains supplementary material, which is available to authorized users.

## Background

Since the emergence of large-scale genomic sequencing, in 2002, the analyses of genomes and proteomes begun to be used and have strength, mainly in recent years. However, it was noticed that there was an exponential increase of more sequences to be deposited resulting in the need to create large databases to store such information which we call Big Data [1]. Currently works highlight the importance of the study of large clusters: as in the prediction of structural families, identifying biologically relevant molecular features in large-scale omics experiments with variable measurements at multiple conditions and to detect in the expansion of the network of interaction between groups and subgroups of biological sequences [2–4]. Clustering methods are essentials for partitioning biological samples and are useful in minimizing the complexity of needed information in extensive datasets [5] and in bioinformatics is the first strategy to search information in biological datasets. In addition, as the size of large biological databases is extensively larger - billions of sequences are currently available for analysis - clustering algorithms generate large number of clusters and superclusters which makes manual curation of these impracticable [6] – i.e. UniRef consortium contains clusters with more than 302,000,000 clusters [7]. Most methods apply the same approach: First, the similarity is calculated and then used to group objects - e.g., experimental samples or biological sequences - into clusters, however the clustering output is useful only if the clusters correspond to the biologically relevant data features that were not used to define the grouping [8]. Currently, two tools are considered as “golds standards” in the clustering sequences to minimize redundancy in large proteins dataset: CD-HIT [9] and UCLUST [10]. CD-HIT is one of the most popular tools and is the state-of-art method [11]. UCLUST is a tool used by thousands of users around the world as high-performance clustering considered faster than the CD-HIT algorithm [12]. However, those tools use greedy strategies for clustering. Furthermore CD-HIT does not support values lower than 40% of similarity and in lower identities whereas UCLUST degrades the quality of alignment [13]. It is also worth pointing out that both the CD-HIT and UCLUST tools require a manual preprocessing step in which the data to be rotated by the algorithms must be organized in order of sequence size, because both algorithms select the largest to minor sequences to choose the representative sequence to the group and align the others from them, not being a random process. Therefore, both CD-HIT and UCLUST are not reliable choices for clustering in large datasets with values less than 30% of similarity so trivial to search sequences with homologies in remotely structures [14]. The most efficient techniques for this prediction use as gold standard the Basic Local Alignment Search Tool (BLAST) ‘all-against-all’ or, in another cases, Markov Clustering (MCL) method adaptations [15]. However, these tools are dependents on alignment metrics requiring a lot of processing and time to generate results mainly in large datasets [16–18].

Alignment-free methods are strong alternatives to alignment-dependent techniques and are also efficient in minimizing the redundancy of biological data its computationally fast and use less memory compared to alignment-based methods [19]. A method that has been highlighting among the clustering techniques of large databases to solve the main time and memory bottlenecks of existing clustering the algorithms is MMSeqs2-Linclust, a deep clustering approach [20]. This method explores the alignment-free analyses and apply two main steps to clustering: the global Hamming distance and the gapless local alignment extending the k-mer match. Sequence pairs are generated under the conditions that satisfying the clustering criteria - e.g., on the E-value, sequence similarity, and sequence coverage- and are linked by an edge. In the end, the greedy incremental algorithm locates a cluster so that each input sequence has an edge to the representative sequence of its cluster [21]. Ultimately, alignment-free methods have been applied to problems ranging from whole-genome and are particularly useful for processing and analyzing Next-Generation Sequencing (NGS) data. However, the benchmark data sets are required to explore the full potential of alignment-free methods [22].

The validity of the clusters is challenging: information from external clusters are needed because they are not known in advance. At this point, the lack of a priori knowledge about the number of clusters underlying in the dataset makes it indispensable and an efficient metric is necessary to compare clustering solutions with different number of clusters [23]. Validity is constantly being questioned because there is a need for standardization of metrics, besides the application of internal and external metrics and the use of consistent bases of biological value [24]. Another point is the application of a high level of programming skills on the part of researchers to analyze large volumes of data [25]: generally, each tool uses a different output and makes difficult the manipulation of data which hinders the fluidity of the researches [26].

To explore the potential of the alignment-free method associated with a strategy that combines hashes and BCOM matrices to reduce the need for the slow sequence alignments, we have developed the RAFTS^3^G. We incorporated the binary search as an option cluster input criterion to align the best *n* candidates, a new alternative proposal for clustering analyses in proteins sequences data. We compared RAFTS^3^G with three main clustering methods exploring standard metrics applied to database “gold standard” of enzymes family adopting as criterion the default parameters of all methods.

### RAFTS^3^G implementation

To minimize time and maintaining consistency in data analysis with proteins, we developed *Rapid Alignment Free Tool for Sequences Similarity Search to Groups* (RAFTS^3^G) tool. RAFTS^3^G was written in MATLAB v2017a explores the RAFTS3 engineer (Additional file 1: Figure S1) and uses integrates functions, the Bioinformatics Toolbox and an in-house library.

## Results

### The RAFTS^3^G overview

RAFTS^3^G applies as search engine RAFTS3 [27] tool, which purpose is to perform faster by minimizing disk access storing sequences information in RAM and in addition to reducing the need for slow sequence alignments. RAFTS3 has a hashing strategy based on k-mers to directly access sequence data – the sequence itself and the Co-Occurrence Matrix of amino acid residues (BCOM). BCOM are sets of 50 bytes containing a binary matrix within amino-acid sequential co-occurrence data for a given sequence. The comparison between BCOM of two sequences is faster than to alignment them to get similarity metric. When RAFTS3 searches for sequence similarities, however, it allows the user to choose to align a set of the top *n* selected candidates within some k-mer match against to a query sequence. The metric provided by BCOM [27] is effective to sort a set of sequences according to their similarity, the similarity measure based on identities, enabled when alignment is performed, is desirable when the intention is to hold clusters and it is often selected as cut-off criterion [28]. Once aligning every subject candidate would be impeditive to a rapid approach sequence grouping algorithm, we studied ways to minimize the need of alignment in RAFTS^3^G; it will be discussed forward, while we present the algorithm.

From a set of input sequences in a FASTA format - variable or file -, for each sequence not grouped yet, RAFTS^3^G exploits a formatted RAFTS3 data base searching for similar sequences. Candidates are ordered by higher BCOM similarity to the query. To select which from candidates should be in the same cluster of the query sequence, given a cut-off value (RAFTS3 self-score), the user can choose:

i) Align the query with up to a limited n number of the BCOM ordered candidates, living behind the rest.
ii) Make a binary search aligning candidates/query to find the cutting point where all sequences of lower order should be as similar or more than the sequence in this point. Sequences of higher order are likely less similar then the stipulated by the cutoff criterion and are left.

The step in ii) is the only change we made in original RAFTS3 approach in order to program RAFTS^3^G. The main gain of the binary search approach is to allow the constrution of a cluster within less steps, since it finds most sequences related to a query in a single search, aligning only a relatively small number of candidates (O(log_2_(n)).

In both cases we have a list of *sequences to group* that are supposed to be at least as similar to the query as the measure defined in cut-off.

It remains now review the assembled groups based on the sequences to group:

- if the query found already grouped sequences, all the groups found are joined in a single one and all other sequences to group are added in this group;
- if none of the sequence to group is member of a previously created group then a new group is built and these sequences are added to it.

**Fig. 1:**
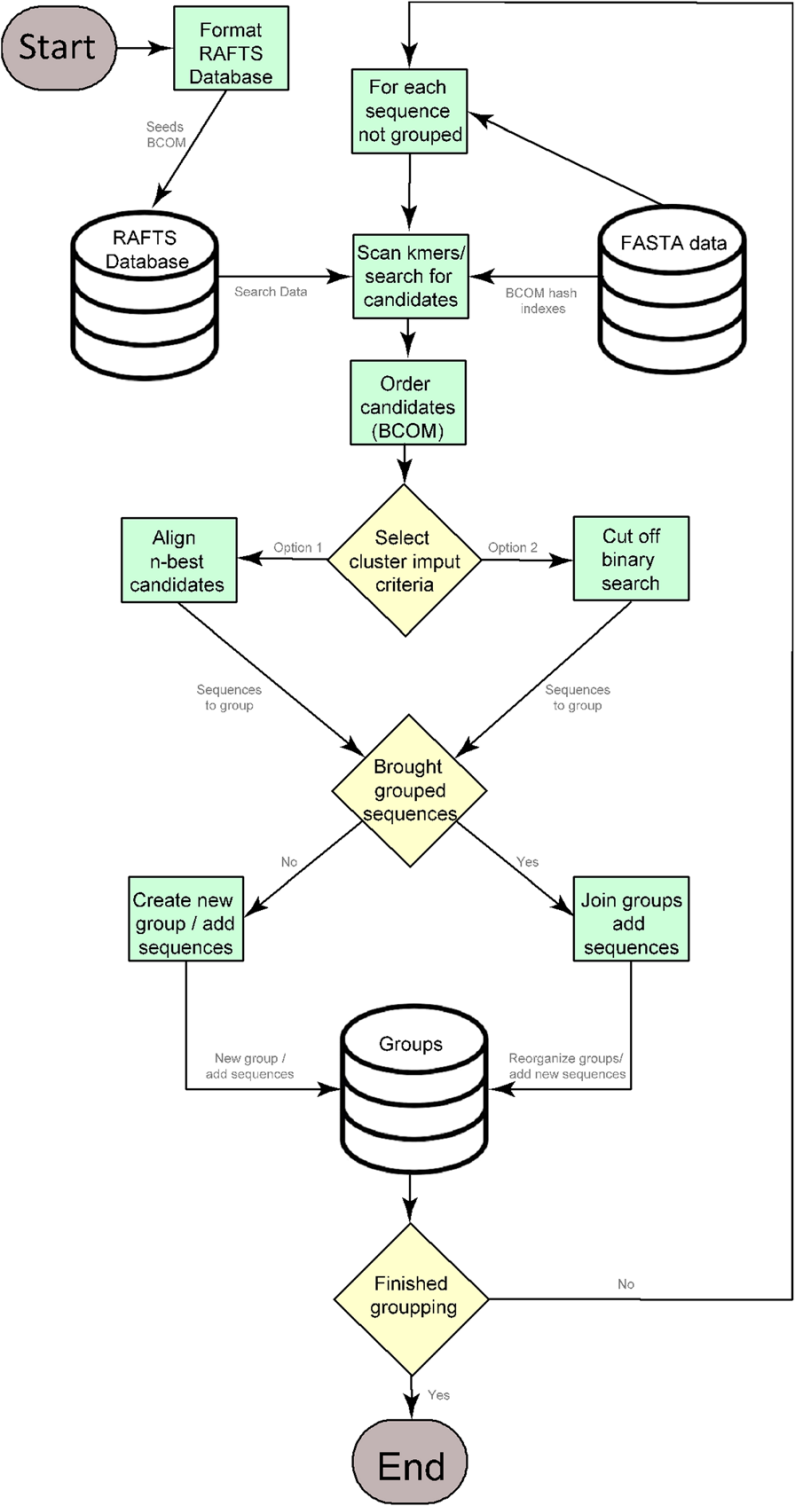
RAFTS3G pipeline: cut-off criteria to candidates selection and the grouping generation. Initially, RAFTS^3^G formats the FASTA file into a seeds of BCOM in RAFTS Database. The search for candidates with k-mer scan from RAFTS Database against a FASTA data indexed into *Hash* BCOM is performed. The candidates are ordered by similarity into a new BCOM matrix which are submitted under a cluster input criteria selection, which may be option 1 -Align *n* sequences candidates- or option 2 – Binary cut-off sequences search. Clustered sequences are available after the selection where groups are joined and sequences are added or if clustered sequences is not accessible a new group is created

While there are sequences to be analyzed these steps will be repeated for each of them. See (Fig. 1). The RAFT^3^G output is easier to be manipulated by the end user because it is in FASTA format with an extra log is generated with clusters information (Additional file 1: Figure S2).

### RAFTS^3^G clustering in large dataset

**Fig. 2:**
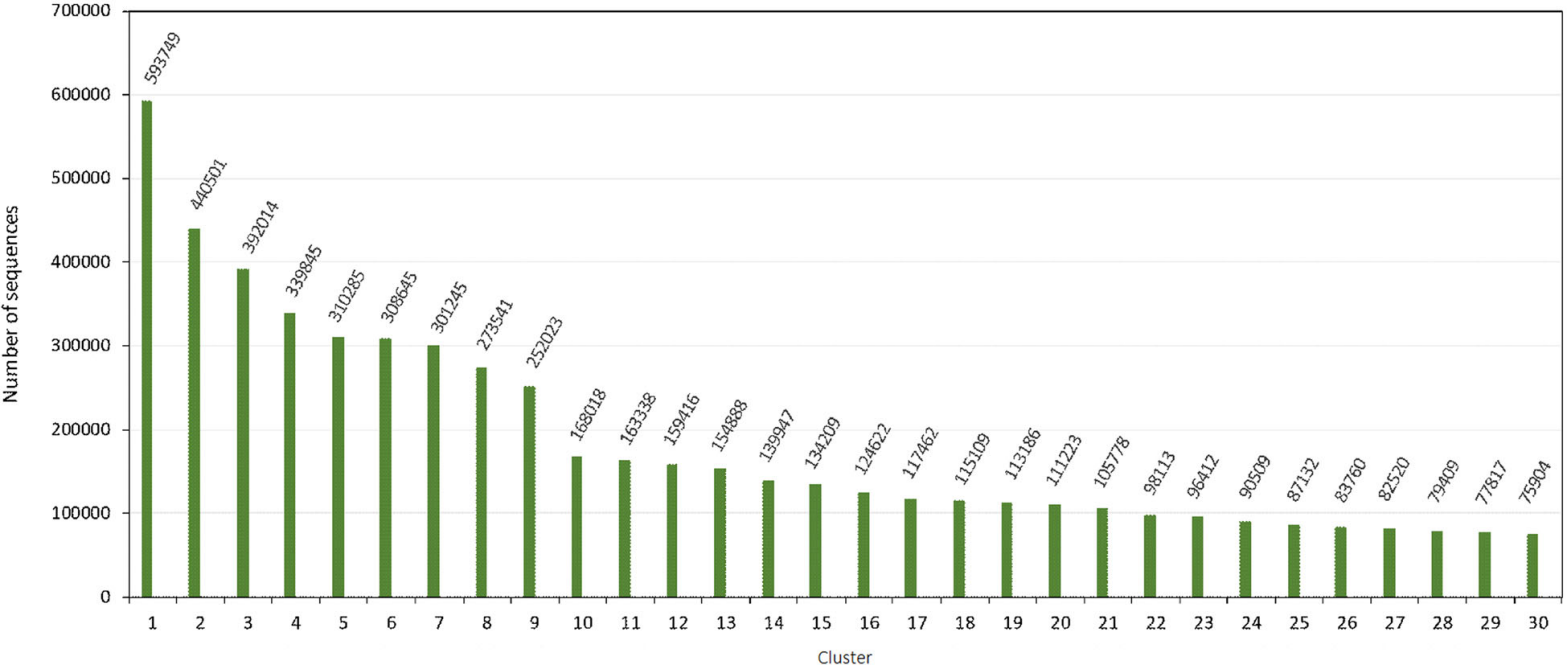
Top 30 clusters (by order number) database generated by RAFTS3G. The majors clusters grouped with RAFTS^3^G in 0.5 similarity threshold using the NR-NCBI database (results available on Additional file 1: Table S3). To performs this test, we adopted Machine 3 configuration (Available on Additional file 1: Table S1)

We performed RAFTS^3^G using the Ref-Seq Non-Redundant protein from NCBI database (NCBI/NR) [29] - with 78,002,046 sequences deposited at this release. We generated 12,594,179 Total clusters of which 4,127,885 are non-unique clusters and 8,466,294 are unique clusters. Twenty-one clusters have more than 100,000 grouped protein sequences and in nine of them exceed 200,000 sequences clustered. In Fig. 2 the 30 largest clusters are represented, according to the number of sequences in each cluster. Therefore, with these results, RAFTS^3^G it is possible to generate clusters in a higher set of data. Due to this large set of data we are evaluating the results obtained allow us to bring more information about the developed clustering techniques in future works.

### Benchmark standardization with F1-score

The choice of a good basis is essential for the reliability of the metrics, so we chose the GOLD/Brown base from ASTRAL/SCOPe [30]. For the validation of clusters, we used F1-Score, an external metric that provides the balance between the accuracy and sensitivity measures [31, 32]. The GOLD database - a collection “gold standard” of enzymes families experimentally validated [33] totalizing 866 sequences - to evaluation of clusters generated for RAFTS^3^G compared to three highlighted methods**.** The Brown database is a collection of experimentally classified enzymes with extreme remote similarities and this database is a challenge to be correclty grouped because extreme remote similarities sequences have low identity which generates many false positives in the clustering process [14]. In comparison with CD-HIT we exemplifying this difficult evaluated the F1-Score, accuracy and sensibility metrics (Additional file 1: Table S5) and we are improving the RAFTS^3^G to obtain more hits with these data sets. We analysed RAFTS^3^G in 0.5 of similarity threshold in 3 representative clusters from Swissprot/UniProtKB with remote similarity: Apolipoprotein C-IV, Period circadian protein and Ribulose bisphosphate carboxylase/oxygenase activase. We generated the distance matrix calculing the sequences alignments to each cluster and we found that RAFTS^3^G had grouped sequences with great distances and no false positives (Additional file 1: Figure S4). These suggests that RAFTS^3^G was able to group distance sequences with low similarities.

According to the results obtained with GOLD database, in low similarities, between 0.2–0.4 intervals of threshold, RAFTS^3^G presents sensitivity above the other compared tools but without significance. We noticed that all tools seem to have similar performance in similarity of 0.3 - excepts CD-HIT because does not generate groups with this threshold. From the cut-off lines between 0.4 and 0.9 of similarity, we observed the ability of RATS^3^G to group consistent sequences compared to MMSeqs2 (Linclust algorithm) - method which stands out in relation the others two tools Usearch (Uclust algorithm) and CD-HIT. As all the methods compared are developed to reduce redundancy, in the higher similarities between the values of 0.8–0.9 of similarity we observed an equity between the results obtained between MMSeqs, USEARCH and CD-HIT. In this range RAFTS^3^G has a 10% gain of F1-Score in relation to the others. (Comparison with CD-HIT and UCLUST performed against Astral/SCOPe of proteins database in 20 to 90% of similarity is available at Additional file 1: Table S2 and S4).

**Fig. 3:**
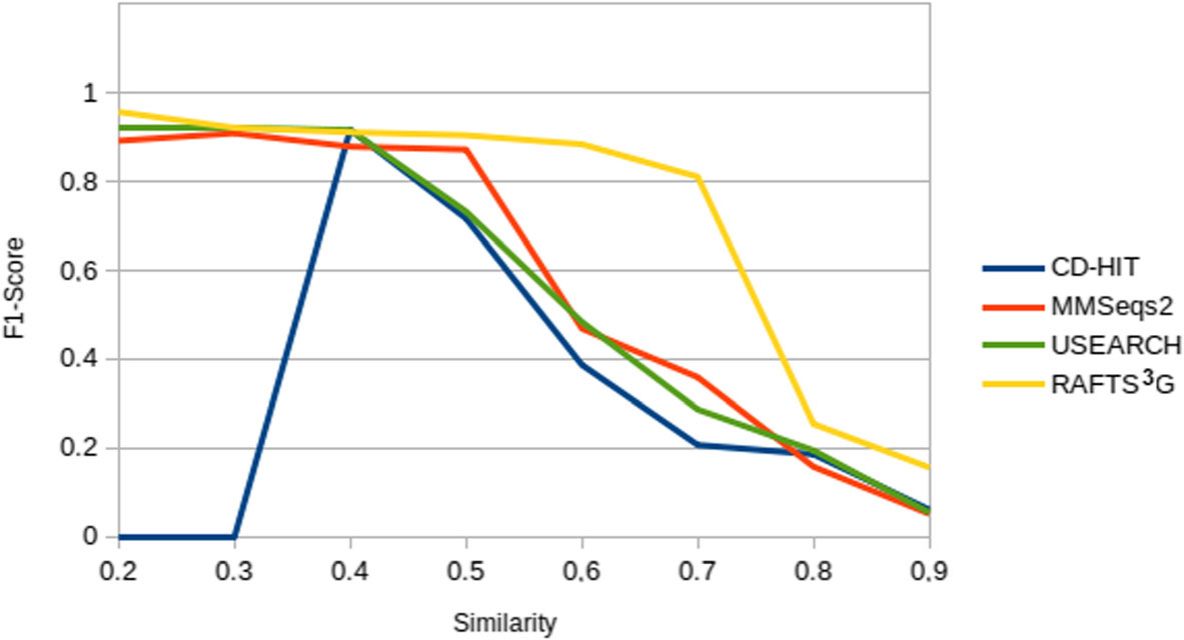
F1-Score benchmark results in RAFTS3G, MMSeqs2 (Linclust), CD-HIT and USEARCH (UCLUST) softwares. The tools were evaluated by running the GOLD database of ASTRAL/SCOPe in the similarity of 0.2 to 0.9, with a range of 0.1, and the F1-Score (families as reference) was calculated for the results (Additional file 1: Table S6). The four methods were run with recommended parameters in the available user documentation (Available on Additional file 1: Figure S3a)

Analyzing these points, RAFT^3^G is the best choice optimized to be more permissible to members inclusion when the clusters increase (Fig. 3). This is interesting when the user wants to “guess” or to “risk” a data set when the similarity does is not known by user. Other methods generate more restricted clusters and choose to lose these informations. In metagenome data, for example, where the collected material is very heterogeneous and abundant, using a strategy which increases sensitivity or probability of clustering sequences mainly at an early stage of data mining is crucial to the success of the experimentation and analysis.

### Binary search input criteria

**Fig. 4:**
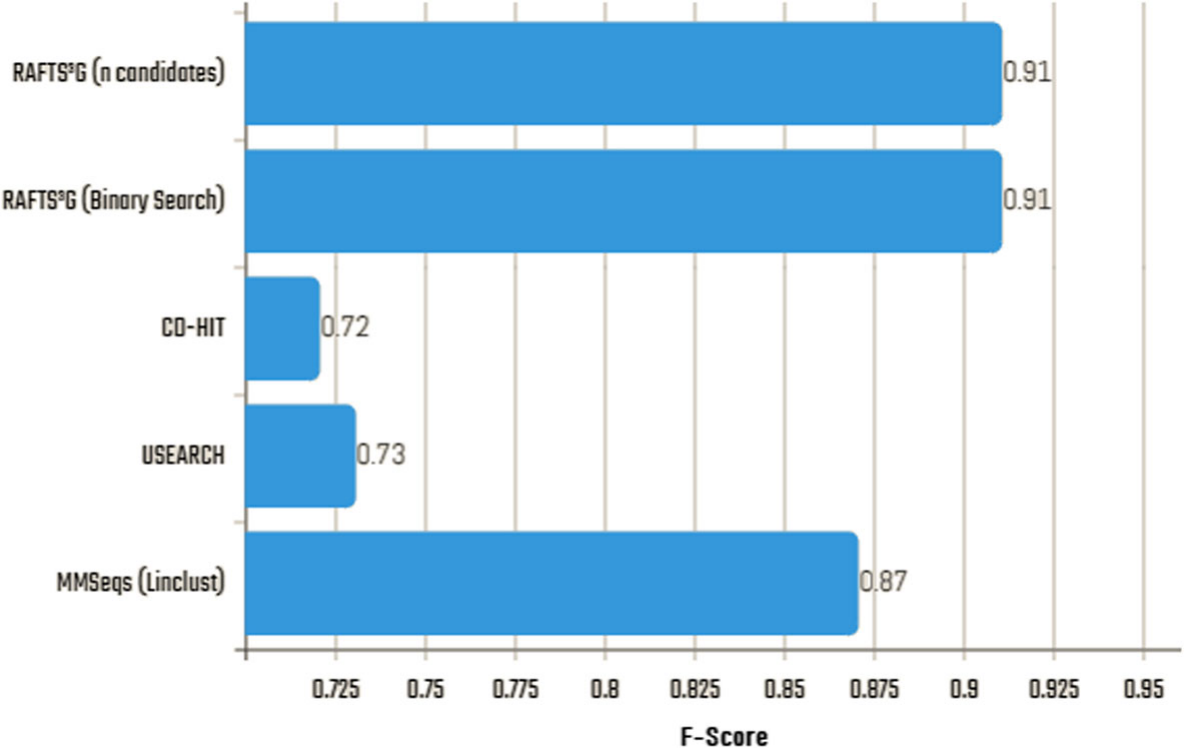
F1-Scores from clustering methods comparison with RAFTS3G binary search and RAFTS3G n candidates. No significative variance was detected in RAFTS^3^G using binary search – performed using 0.5 cut-off – compared with RAFTS^3^G *n* candidates to clustering sequences. The result reflects the F1-Score mean parameter for four tools. The softwares were run with the parameters recommended in users’ documentation presented by each author (Available on Additional file 1: Figure S3b e c)

In the RAFTS^3^G overview, we bring the proposal of a binary search to the assembly of the clusters after the selection of the candidates obtained by the RAFTS3 engineering, instead of the cut-off for the groups to be based on the alignment of the sequences by the selection of *n* candidates. Results of clusters generated with the GOLD base (Astral / SCOPe) suggest that this type of strategy maintains the sensitivity / accuracy ratio (Fig. 4). In addition to being significantly high - around 91% of F1-Score for RAFTS^3^G in relation to 0.87 in MMSeqs, 0.73 of USEARCH and 0.72 of CD-HIT - another observable advantage is in reducing time - binary search reduced by up to 73% of the overall execution time of RAFTS^3^G - maintaining the quality of the data generated.

## Conclusions

The goal of this study is to provide an alternative to clustering analyses with reduced losses of biological data information improving the alignment-free concept. RAFTS^3^G is able to group up to millions of sequences. Furthermore, we brought a benchmark analysis using the F1-score as an external metric to evaluate the performance of the main clustering methods by exploring a wide range of similarity and found that the RAFTS^3^G strategy is the best optimized - to be more permissive - which reflects in greater accuracy and sensitivity in generating clusters with consistent biological content. The binary search input criteria for creating groups demonstrates to be efficient to create or to integrate candidate groups as the overall alignment of *n* candidates.

We hope the RAFTS^3^G algorithm will prove helpful to assist the researcher to explore the widest range of available data and to make them more consistent.

### Data and RAFTS^3^G availability

**Project name**: RAFTS^3^G.

**Project Home Page**: https://sourceforge.net/projects/rafts-g/

**Operating System**: Windows and Linux (× 86 and × 64 versions).

**Programming Language**: Designed in Matlab® v2012.

**Other requirements:** MCR runtime (v7.17) is required to runs.

**License**: the software is under licensed by Matlab® v 2012.

**Any restrictions to use by non-academics:** none.

## Additional file

Additional file 1: Support material - system requirements, extra information about RAFTS3 engineering, methodology overflow, tests, additional links and literatures. (DOCX 808 kb)

## Data Availability

The RAFT^3^G is freely accessible and can be downloaded without user registration at: https://sourceforge.net/projects/rafts-g/ and additional informations in supplementary material.
