# Supplementary material for: RAFTS3G: an efficient and versatile clustering software to analyses in large protein datasets
Source: BMC Bioinformatics. 2019 Jul 15;20:392. doi: 10.1186/s12859-019-2973-4 (PMC6631606; doi:10.1186/s12859-019-2973-4)
Supplement: Supplementary file 1 — Support material - system requirements, extra information about RAFTS3 engineering, methodology overflow, tests, additional links and literatures. (DOCX 808 kb) [file 12859_2019_2973_MOESM1_ESM.docx]

# RAFTS³G: An efficient and versatile clustering software to analyses in large protein datasets

Bruno Thiago de Lima Nichio¹² (brnichio@gmail.com), Aryel Marlus Repula de Oliveira¹ (aryelmarlus@gmail.com), Camilla Reginatto de Pierri¹² (camilla_kaori@hotmail.com), Leticia Graziela Costa Santos¹ (grazielalgs@gmail.com), Alexandre Quadros Lejambre¹ (lejambre@gmail.com), Ricardo Assunção Vialle¹ (ricardovialle@gmail.com), Nilson Antônio da Rocha Coimbra¹ (nilson.coimbra@gmail.com), Dieval Guizelini¹ (dieval@terra.com.br), Jeroniza Nunes Marchaukoski¹ (jeroniza@gmail.com), Fabio de Oliveira Pedrosa¹² (fpedrosa@ufpr.br) and Roberto Tadeu Raittz¹ (raittz@ufpr.br)

¹Laboratory of Bioinformatics, Professional and Technical Education Sector from the Federal University of Paraná, Curitiba, PR, Brazil

^2^Department of Biochemistry, Biological Sciences Sector – Federal University of Paraná (UFPR), Curitiba, PR, Brazil

# Materials and methods details

# 1 Machine specifications:

| Machine 1 | Lenovo ThinkCentre M90P Desktop |
| --- | --- |
| Processor | Intel(R) Core (TM) i5 CPU 650 3.20GHz Cache 4MB |
| emory Size: | 15 GB SODIMM DDR3 |
| Operating System: | Biolinux 8.0 x86_x64 (based on Ubuntu 18.04.01 LTS) |
| Machine 2 | HP Pavillon DM4-2075 Notebook |
| Processor: | Intel(R) Core (TM) i5 CPU 2410M 2.30GHz Cache 3MB |
| Memory Size: | 4 GB SODIMM DDR3 |
| Operating System: | Windows 10 Pro x64 |
| Machine 3 | Lenovo Server |
| Processor: | 40-core Intel Xeon |
| Memory Size: | 256 GB SODIMM DDR3 |
| Operating System: | Ubuntu 16.04.01 LTS |

**Table S1:** *The machine parameters performed in our tests.* The Machine 1 is a desktop used to compare clustering methods. Machine 2 is a laptop used to measure RAFTS³G performance with low configurations to simulate the user consumption. Machine 3 is a Server from bioinformatics laboratory to clustering NR-NCBI Database.

**2** **RAFTS3 workflow**

RAFTS³ (available at https://sourceforge.net/projects/RAFTS³/) is a tool that searches for similarities between protein sequences and that also uses a filter *Hash* function for the selection of candidates is based on *k-mers* shared and a measure of comparison using a co-occurrence matrix of amino acid residues (BCOM) [1]. RAFTS³G (available at <https://sourceforge.net/projects/rafts-g/>) was developed as an application of the RAFTS³ tool for grouping of homolog sequences (**Figure 1**). The script creates a base in which the query is performed by the RAFTS³ engine. Each protein in the input file is evaluated, initially, as a group, and the result of the query to the Bank assesses the similarity between the proteins by the value of self-score. homolog groups are formed on the basis of the verification of the number of cases recognized by the minimum similarity assessed. Additional information is more detailed in RAFTS3 repository. The Matlab Compiler Runtime (MCR) v7.17 is need to runs RAFTS3 and RAFTS³G ( available at: <https://www.mathworks.com/products/compiler/matlab-runtime.html>)


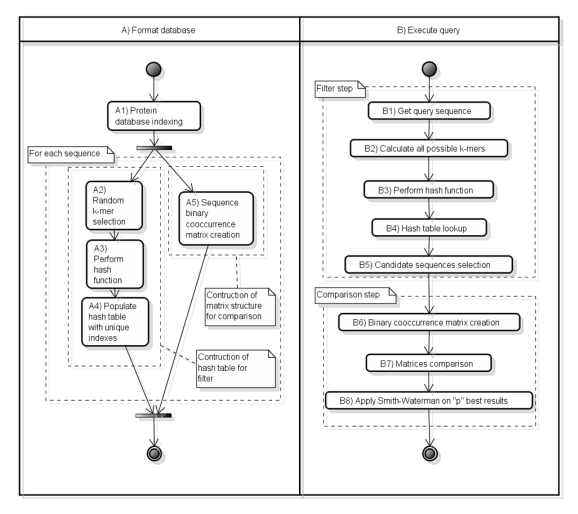


**Figure S1**. *RAFTS³ flowchart: format database and query search overview processes*. A) Shows the database formatting processes, which involve construction of two structures used in query sequence search, a hash table and a set of binary cooccurrence matrices. B) Shows the process for searching and comparison of a query sequence, with filtering and comparison steps separated.


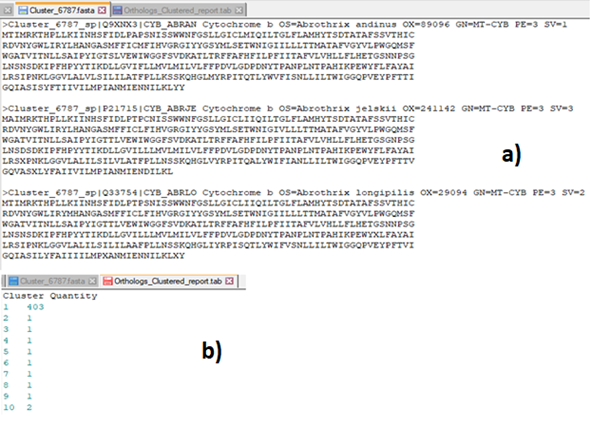


# Figure S2. *The RAFTS³G outputs formats.* a) An example of RAFTS³G clustered sequences in FASTA format. To each sequence is added a respective cluster number. b) The clustered report in tab file relating the Cluster number and the quantity of clustered sequence generated by RAFT³G.

**3 Methodology Overflow**


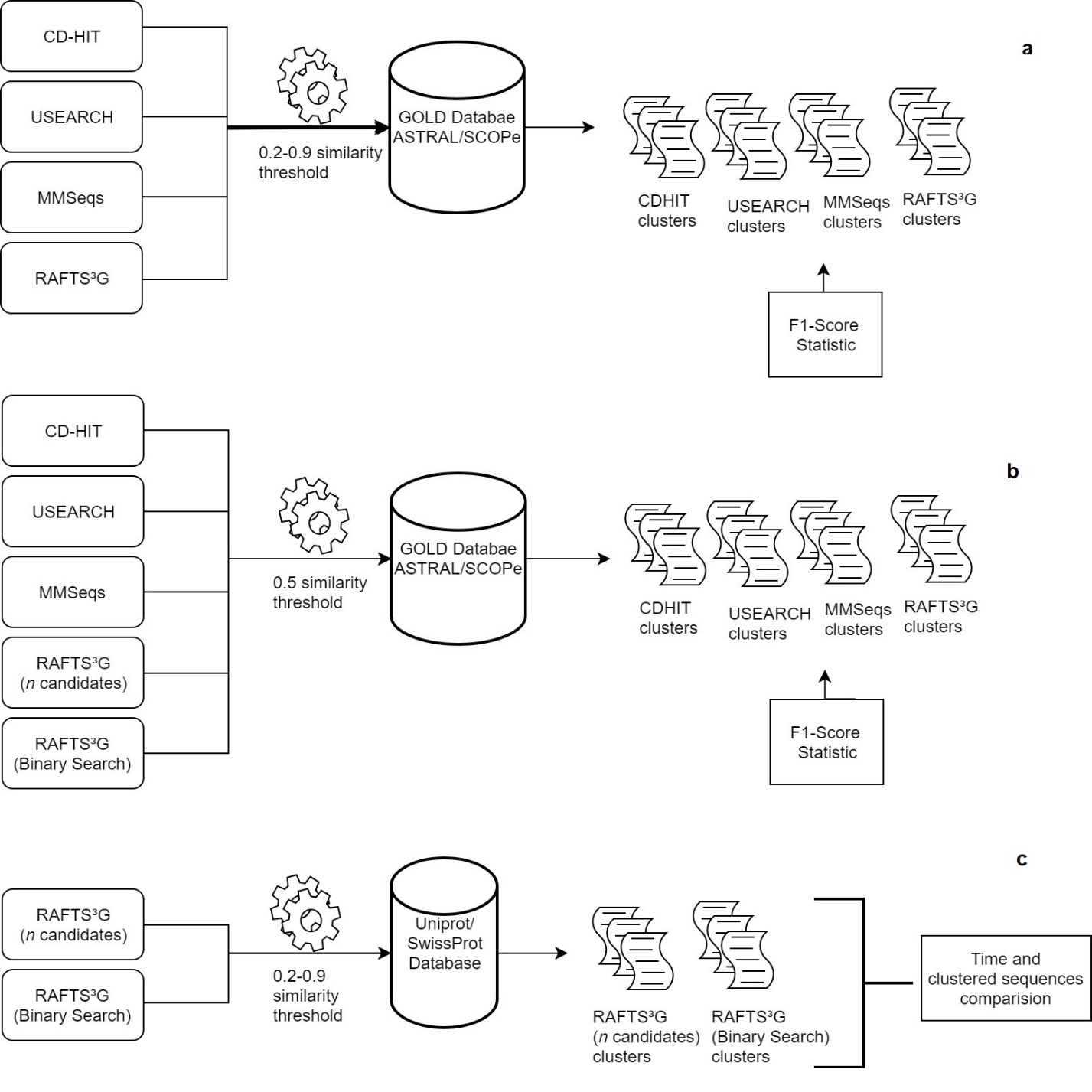


**Figure S3**. *Methodology Overview in workflow.* **a** clustering methods F1-Score benchmark **b** to Binary Search input criteria evaluation in RAFTS³G compared with clustering methods and **c** to Binary Search performance evaluation in RAFTS³G on/off (*n* candidates).

**4 RAFTS³G comparison with CD-HIT and UCLUST performed against Astral/SCOPe of proteins database in 20% to 90% of similarity**

The *Structural Classification of Proteins — extended* (SCOPe) compendium (available at: http://scop.berkeley.edu/) provides databases useful for analyzing protein structures and their sequences. Through a combination of automation and manual curation, and corrects some errors, aiming to have the same accuracy as the fully hand-curated SCOP releases [2].

SCOPe also incorporates and updates the Astral database. We have performed the tests with GOLD database - release 2.07 stable version available on March 2018. The GOLD is considered a “gold” database because it has experimentally defined enzyme families.

We adopted the following testing protocol:

- We made several clusters using between 20% to 90% of similarity degree;
- We record the best F1-Score in relation to the cured families and presented the most relevant external metrics.

| Similarity | CD-HIT  (F1-Score) | MMSeqs2  (F1-Score) | RAFTS3G  (F1-Score) | USEARCH  (F1-Score) |
| --- | --- | --- | --- | --- |
| 0.2 | 0* | 0.8921 | 0.9573 | 0.9232 |
| 0.3 | 0* | 0.9093 | 0.9217 | 0.9206 |
| 0.4 | 0.9174 | 0.8789 | 0.9116 | 0.9164 |
| 0.5 | 0.7167 | 0.8725 | 0.9047 | 0.7324 |
| 0.6 | 0.3869 | 0.4688 | 0.8844 | 0.484 |
| 0.7 | 0.207 | 0.3593 | 0.8109 | 0.2871 |
| 0.8 | 0.1861 | 0.158 | 0.2543 | 0.1947 |
| 0.9 | 0.0608 | 0.0516 | 0.1563 | 0.0546 |
| Mean | 0.3093625 | 0.5738125 | 0.72515 | 0.564125 |
| Median | 0.19655 | 0.67065 | 0.89455 | 0.6082 |
| Standard Deviation | 0.342367985 | 0.358329571 | 0.324594472 | 0.356105292 |

**Table S2**. *F1-Score results for CD-HIT, MMseqs2, RAFTS³G and USEARCH clustering softwares*. The results obtained set up the softwares in 0.2 up to 0.9 range of similarity threshold. * CD-HIT does not support similarity lower than 0.4 in machine 2 configurations.

**5 Clustering NCBI Non-Redundant data sequences of proteins database by RAFTS³G in 50% of similarity**

We have selected NR/NCBI database (with 78,002,046 sequences deposited) to performed RAFTS³G. We generated 12,594,179 Total clusters being 4,127,885 clusters Non-unique and 8,466,294 unique clusters.

| Cluster Order | Number of Sequences |
| --- | --- |
| 1 | 593749 |
| 2 | 440501 |
| 3 | 392014 |
| 4 | 339845 |
| 5 | 310285 |
| 6 | 308645 |
| 7 | 301245 |
| 8 | 273541 |
| 9 | 252023 |
| 10 | 168018 |
| 11 | 163338 |
| 12 | 159416 |
| 13 | 154888 |
| 14 | 139947 |
| 15 | 134209 |
| 16 | 124622 |
| 17 | 117462 |
| 18 | 115109 |
| 19 | 113186 |
| 20 | 111223 |
| 21 | 105778 |
| 22 | 98113 |
| 23 | 96412 |
| 24 | 90509 |
| 25 | 87132 |
| 26 | 83760 |
| 27 | 82520 |
| 28 | 79409 |
| 29 | 77817 |
| 30 | 75904 |

**Table S3.** *Top 30 clusters result for clustering with NCBI/NR using RAFTS³G*. The results were obtained with RAFTS³G with 0.5 similarity threshold in NR/NCBI.

**6 Binary Search benchmark**

| Software | F1-Score |
| --- | --- |
| *RAFTS³G (n candidates)* | 0.91 |
| *RAFTS³G (Binary Search)* | 0.91 |
| *CD-HIT* | 0.72 |
| *USEARCH* | 0.73 |
| *MMSeqs (Linclust)* | 0.87 |

**Table S4**. *F1-Score results for clustering methods with RAFTS³G binary search cut-off criteria mode on and off*. All methods were tested using the GOLD Database from ASTRAL/SCOPe adopting 0.5 similarity threshold in the softwares default parameters.

**7 RAFTS³G in clustering of sequences with remote similarities**

Clustering tools have low sensitivity in grouping sequences with remote similarity [3]. In attention to this, we observed the behavior of RAFTS³G in 0.5 of similarity threshold in groups of proteins with remote similarity, for this we selected from the SwissProt/UniProtKB database 20 proteins of 3 representative clusters with remote similarity: Apolipoprotein C-IV, Period circadian protein and Ribulose bisphosphate carboxylase/oxygenase activase.

We calculated the pairwise distance between sequences generating a square distance matrix using the *seqpdist* function (<https://www.mathworks.com/help/bioinfo/ref/seqpdist.html>). This function returns a vector containing biological distances (*d*) between each pair of sequences of a distance matrix based on the mathematical equation (for amino acids):

*d* = -19/20 log(1-p * 20/19).

The character vector or string specifying the scoring matrix to use for the alignment. Choices for amino acid sequences are on BLOSUM50. With this it was possible identifying the similarities between intracluster sequences (**Figure 4**). The lower values of the matrix reveal the more distant or less similarities between the sequences intracluster and 1 for the proteins in relation to itself (**Appendix**).

We identifying the range of sequences with low intracluster similarities: 0.033646 - 1 to Apolipoprotein C-IV, 0.0691 – 1 to Ribulose bisphosphate.

**
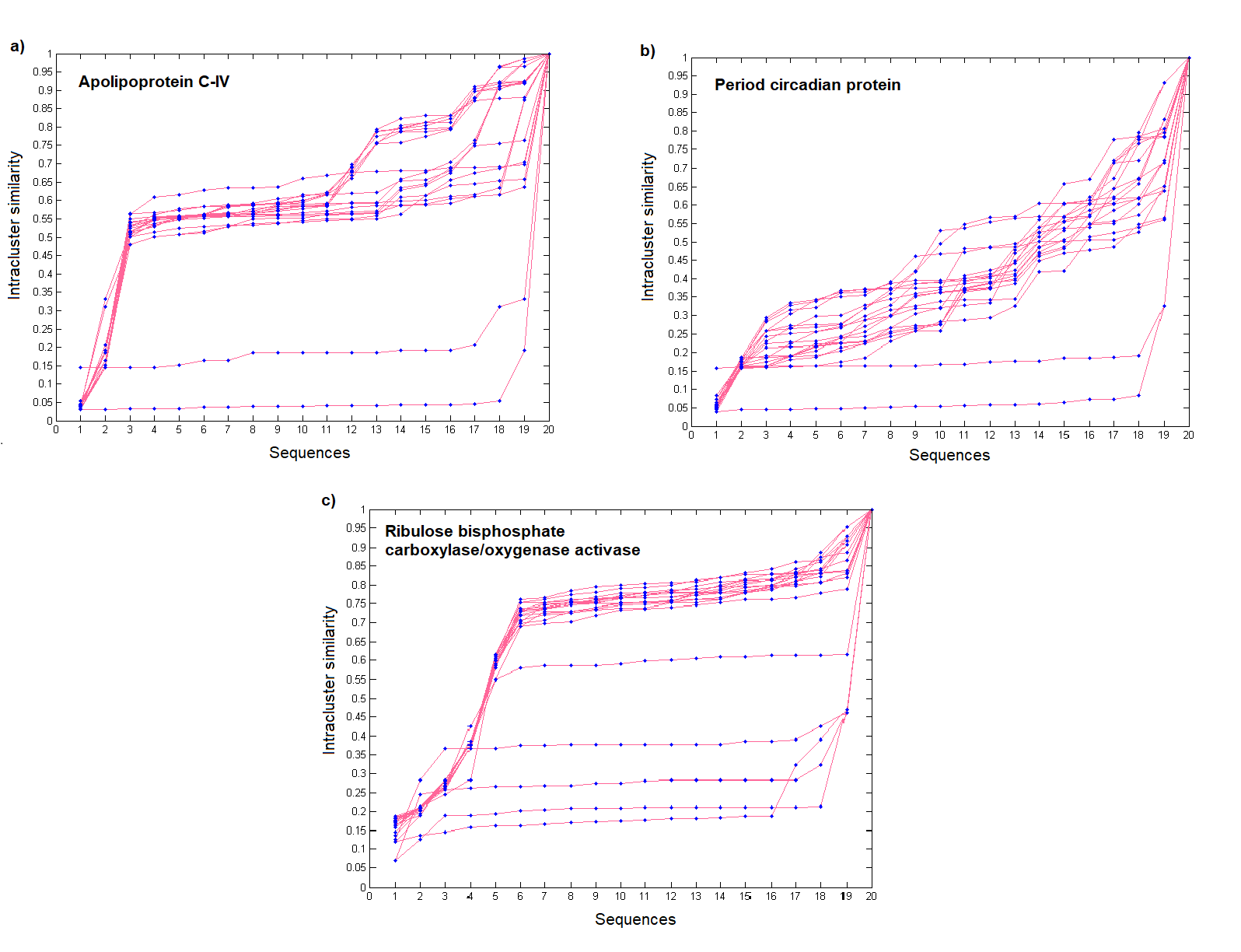
**

**Figure S4**. *Distance distribution in clustered sequences with remote similarities by RAFTS³G*. **a)** Apolipoprotein C-IV, **b)** Period circadian protein and **c)** Ribulose bisphosphate carboxylase/oxygenase activase. We selected the 20 protein sequences to each protein family. The low similarities intracluster between the grouped sequences (~0.05) suggest that low-similarity sequences were grouped.

In parallel, we had made some tests using the Brown database of ASTRAL/SCOPe (<http://scop.berkeley.edu/astral/>) which is caracterized to have 2 datasets of experimentally classified enzymes with extreme remote similarities: 1) 0.5% up to 40% of similarity containing 4493 true classes and 2) 0.5% up to 95% containing 4735 true classes. We adopted the same configurations used in GOLD database to obtain our results using the F1-Score. Here we left some results below to better exemplify the RAFTS³G comparing with the CD-HIT results:

| ASTRAL SCOPe DB | **Tool** | **F1-Score** | **Accuracy** | **Sensitivy** |
| --- | --- | --- | --- | --- |
| **Up to 40% similarity** | **RAFTS³G (0.5)** | **0.0000** | **0.6429** | **0.0000** |
| Up to 40% similarity | CD-HIT (0.5) | 0.0000 | 0.4138 | 0.0000 |
| **Up to 95% similarity** | **RAFTS³G (0.5)** | **0.3062** | **0.6556** | **0.1998** |
| Up to 95% similarity | CD-HIT (0.5) | 0.1670 | 0.6547 | 0.0957 |

**Table S5**. *F1-Score to clustering results by RAFTS³G and CD-HIT in 0.5 of threshold with the Brown database from ASTRAL/SCOPe*.

**8 Clustering UniProtKB/Swiss-Prot database by RAFTS³G with on/off binary search**

| **Self-score** | Unique  Clusters | Clusters  non-Unique | Total  Clusters | RAFTS³G¹  Clustering Time (Hours) | RAFTS³G²  Clustering Time (Hours) |
| --- | --- | --- | --- | --- | --- |
| **20** | 42,479 | 14,250 | 56,999 | 9.8 | 2.42 |
| **30** | 50,395 | 18,976 | 69,371 | 10.4 | 2.51 |
| **40** | 61,737 | 27,089 | 88,826 | 10.5 | 2.67 |
| **50** | 76,920 | 36,612 | 113,532 | 10.6 | 2.75 |
| **60** | 99,474 | 49,815 | 149,289 | 11.1 | 3.16 |
| **70** | 129,779 | 60,049 | 189,828 | 11.5 | 3.56 |
| **80** | 172,476 | 67,854 | 240,330 | 12.7 | 4,10 |
| **90** | 238,093 | 70,589 | 308,682 | 13.9 | 4.87 |

**Table S6**. *Clustering result by RAFTS³G algorithm using various self-scores metrics on in machine 2 conditions*. RAFTS³G¹ with binary search cut-off mode off ( *n* candidates) and RAFTS³G² with binary search mode on.

# References

[1] Vialle, R. A., Pedrosa, F. O., Weiss, V. A., Guizelini, D., Tibaes, J. H., Marchaukoski, J. N., Raittz, R. T. (2016). RAFTS³: Rapid Alignment-Free Tool for Sequence Similarity Search. *bioRxiv*, 55269. https://doi.org/10.1101/055269.

[2] Fox, N. K., Brenner, S. E., & Chandonia, J. M. (2014). SCOPe: Structural Classification of Proteins - Extended, integrating SCOP and ASTRAL data and classification of new structures. Nucleic Acids Research, 42(D1). https://doi.org/10.1093/nar/gkt1240.

[3] Chen, J., Long, R., Wang, X. L., Liu, B., & Chou, K. C. (2016). DRHP-PseRA: Detecting remote homology proteins using profile-based pseudo protein sequence and rank aggregation. Scientific Reports, 6(August), 1–7. <https://doi.org/10.1038/srep32333>

**Appendix**

**Apolipoprotein C-IV: Distance Matrix obtained with *seqpdist***

|  | **\|P0DP53\|** | **\|P0DKW1\|** | **\|P0DKW2\|** | **\|Q3SYR5\|** | **\|P0DOC4\|** | **\|P0DKU6\|** | **\|P55056\|** | **\|Q61268\|** | **\|P0DML4\|** | **\|P0DMN8\|** | **\|A0A096P2H6\|** | **\|P0DKY3\|** | **\|P0DML5\|** | **\|P0DKW3\|** | **\|P55057\|** | **\|P55797\|** | **\|P0DKW4\|** | **\|P0DML6\|** | **\|Q1D5X9\|** | **\|P85428\|** |
| --- | --- | --- | --- | --- | --- | --- | --- | --- | --- | --- | --- | --- | --- | --- | --- | --- | --- | --- | --- | --- |
| **\|P0DP53\|** | 1 | 0,513054 | 0,52446 | 0,652694 | 0,613771 | 0,558214 | 0,532899 | 0,501189 | 0,645757 | 0,608222 | 0,550173 | 0,549282 | 0,658798 | 0,528409 | 0,537341 | 0,544955 | 0,531818 | 0,640038 | 0,039122 | 0,144843 |
| **\|P0DKW1\|** | 0,513054 | 1 | 0,897732 | 0,572242 | 0,528805 | 0,788403 | 0,774337 | 0,563634 | 0,561491 | 0,697313 | 0,796384 | 0,797324 | 0,588588 | 0,925086 | 0,560458 | 0,584989 | 0,90352 | 0,555522 | 0,039933 | 0,151712 |
| **\|P0DKW2\|** | 0,52446 | 0,897732 | 1 | 0,564701 | 0,537815 | 0,7947 | 0,758151 | 0,551126 | 0,561709 | 0,668935 | 0,78703 | 0,787994 | 0,57671 | 0,910126 | 0,571932 | 0,586327 | 0,919228 | 0,555718 | 0,03831 | 0,186262 |
| **\|Q3SYR5\|** | 0,652694 | 0,572242 | 0,564701 | 1 | 0,656573 | 0,622452 | 0,614641 | 0,567161 | 0,748166 | 0,68037 | 0,615857 | 0,620275 | 0,763455 | 0,583989 | 0,585704 | 0,594186 | 0,582881 | 0,755928 | 0,033646 | 0,192334 |
| **\|P0DOC4\|** | 0,613771 | 0,528805 | 0,537815 | 0,656573 | 1 | 0,550428 | 0,50718 | 0,480658 | 0,688195 | 0,56338 | 0,540425 | 0,538439 | 0,704387 | 0,544958 | 0,500463 | 0,515634 | 0,548287 | 0,674229 | 0,053104 | 0,1649 |
| **\|P0DKU6\|** | 0,558214 | 0,788403 | 0,7947 | 0,622452 | 0,550428 | 1 | 0,87936 | 0,604045 | 0,561683 | 0,689943 | 0,964308 | 0,963816 | 0,592717 | 0,822807 | 0,581214 | 0,611982 | 0,803828 | 0,555875 | 0,045021 | 0,185689 |
| **\|P55056\|** | 0,532899 | 0,774337 | 0,758151 | 0,614641 | 0,50718 | 0,87936 | 1 | 0,56281 | 0,571039 | 0,659444 | 0,871974 | 0,877157 | 0,586994 | 0,793177 | 0,547219 | 0,594902 | 0,755033 | 0,565213 | 0,044456 | 0,192719 |
| **\|Q61268\|** | 0,501189 | 0,563634 | 0,551126 | 0,567161 | 0,480658 | 0,604045 | 0,56281 | 1 | 0,506442 | 0,616229 | 0,589212 | 0,588268 | 0,528053 | 0,552799 | 0,611987 | 0,872967 | 0,549358 | 0,511342 | 0,041356 | 0,310852 |
| **\|P0DML4\|** | 0,645757 | 0,561491 | 0,561709 | 0,748166 | 0,688195 | 0,561683 | 0,571039 | 0,506442 | 1 | 0,634513 | 0,561542 | 0,559523 | 0,919967 | 0,56435 | 0,552964 | 0,552644 | 0,56875 | 0,978665 | 0,031659 | 0,144082 |
| **\|P0DMN8\|** | 0,608222 | 0,697313 | 0,668935 | 0,68037 | 0,56338 | 0,689943 | 0,659444 | 0,616229 | 0,634513 | 1 | 0,689997 | 0,691033 | 0,676383 | 0,681475 | 0,637455 | 0,633777 | 0,678145 | 0,628994 | 0,042146 | 0,144287 |
| **\|A0A096P2H6\|** | 0,550173 | 0,796384 | 0,78703 | 0,615857 | 0,540425 | 0,964308 | 0,871974 | 0,589212 | 0,561542 | 0,689997 | 1 | 0,985176 | 0,592621 | 0,83085 | 0,587726 | 0,599629 | 0,81184 | 0,555718 | 0,042563 | 0,185738 |
| **\|P0DKY3\|** | 0,549282 | 0,797324 | 0,787994 | 0,620275 | 0,538439 | 0,963816 | 0,877157 | 0,588268 | 0,559523 | 0,691033 | 0,985176 | 1 | 0,59055 | 0,831738 | 0,58566 | 0,598654 | 0,812759 | 0,553708 | 0,044171 | 0,185689 |
| **\|P0DML5\|** | 0,658798 | 0,588588 | 0,57671 | 0,763455 | 0,704387 | 0,592717 | 0,586994 | 0,528053 | 0,919967 | 0,676383 | 0,592621 | 0,59055 | 1 | 0,583759 | 0,592035 | 0,560956 | 0,59038 | 0,911135 | 0,032625 | 0,144153 |
| **\|P0DKW3\|** | 0,528409 | 0,925086 | 0,910126 | 0,583989 | 0,544958 | 0,822807 | 0,793177 | 0,552799 | 0,56435 | 0,681475 | 0,83085 | 0,831738 | 0,583759 | 1 | 0,556572 | 0,583396 | 0,922727 | 0,558378 | 0,040861 | 0,186227 |
| **\|P55057\|** | 0,537341 | 0,560458 | 0,571932 | 0,585704 | 0,500463 | 0,581214 | 0,547219 | 0,611987 | 0,552964 | 0,637455 | 0,587726 | 0,58566 | 0,592035 | 0,556572 | 1 | 0,615645 | 0,566754 | 0,560181 | 0,032842 | 0,206765 |
| **\|P55797\|** | 0,544955 | 0,584989 | 0,586327 | 0,594186 | 0,515634 | 0,611982 | 0,594902 | 0,872967 | 0,552644 | 0,633777 | 0,599629 | 0,598654 | 0,560956 | 0,583396 | 0,615645 | 1 | 0,576612 | 0,557663 | 0,037136 | 0,330794 |
| **\|P0DKW4\|** | 0,531818 | 0,90352 | 0,919228 | 0,582881 | 0,548287 | 0,803828 | 0,755033 | 0,549358 | 0,56875 | 0,678145 | 0,81184 | 0,812759 | 0,59038 | 0,922727 | 0,566754 | 0,576612 | 1 | 0,562792 | 0,036514 | 0,186227 |
| **\|P0DML6\|** | 0,640038 | 0,555522 | 0,555718 | 0,755928 | 0,674229 | 0,555875 | 0,565213 | 0,511342 | 0,978665 | 0,628994 | 0,555718 | 0,553708 | 0,911135 | 0,558378 | 0,560181 | 0,557663 | 0,562792 | 1 | 0,030115 | 0,164747 |
| **\|Q1D5X9\|** | 0,039122 | 0,039933 | 0,03831 | 0,033646 | 0,053104 | 0,045021 | 0,044456 | 0,041356 | 0,031659 | 0,042146 | 0,042563 | 0,044171 | 0,032625 | 0,040861 | 0,032842 | 0,037136 | 0,036514 | 0,030115 | 1 | 0,192279 |
| **\|P85428\|** | 0,144843 | 0,151712 | 0,186262 | 0,192334 | 0,1649 | 0,185689 | 0,192719 | 0,310852 | 0,144082 | 0,144287 | 0,185738 | 0,185689 | 0,144153 | 0,186227 | 0,206765 | 0,330794 | 0,186227 | 0,164747 | 0,192279 | 1 |

**Period circadian protein: distance matrix obtained with *seqpdist***

|  | **\|P22035\|** | **\|P81012\|** | **\|Q17285\|** | **\|Q03293\|** | **\|Q26288\|** | **\|Q03294\|** | **\|Q03295\|** | **\|Q04535\|** | **\|Q26289\|** | **\|Q25206\|** | **\|Q03296\|** | **\|Q04536\|** | **\|Q04537\|** | **\|Q26287\|** | **\|Q25109\|** | **\|Q25221\|** | **\|Q25435\|** | **\|Q26231\|** | **\|Q26612\|** | **\|Q27135\|** |
| --- | --- | --- | --- | --- | --- | --- | --- | --- | --- | --- | --- | --- | --- | --- | --- | --- | --- | --- | --- | --- |
| **\|P22035\|** | 1 | 0,325617 | 0,04665 | 0,044881 | 0,044872 | 0,073764 | 0,084614 | 0,072535 | 0,046407 | 0,052883 | 0,06114 | 0,064466 | 0,038712 | 0,054669 | 0,056255 | 0,046709 | 0,058641 | 0,053762 | 0,058568 | 0,048837 |
| **\|P81012\|** | 0,325617 | 1 | 0,157082 | 0,163496 | 0,164873 | 0,177273 | 0,17625 | 0,185204 | 0,164563 | 0,191983 | 0,169444 | 0,17459 | 0,161824 | 0,168919 | 0,163191 | 0,164957 | 0,188474 | 0,165152 | 0,164894 | 0,185939 |
| **\|Q17285\|** | 0,04665 | 0,157082 | 1 | 0,174655 | 0,164657 | 0,287421 | 0,282418 | 0,294039 | 0,162947 | 0,324407 | 0,23084 | 0,257861 | 0,160562 | 0,185396 | 0,417886 | 0,523844 | 0,513149 | 0,560201 | 0,538353 | 0,421066 |
| **\|Q03293\|** | 0,044881 | 0,163496 | 0,174655 | 1 | 0,548461 | 0,405747 | 0,412603 | 0,391309 | 0,619976 | 0,191023 | 0,505089 | 0,486054 | 0,637835 | 0,586558 | 0,274496 | 0,242531 | 0,204288 | 0,266014 | 0,239285 | 0,271905 |
| **\|Q26288\|** | 0,044872 | 0,164873 | 0,164657 | 0,548461 | 1 | 0,389342 | 0,395559 | 0,374924 | 0,721874 | 0,22529 | 0,50495 | 0,471673 | 0,550464 | 0,603419 | 0,267271 | 0,230115 | 0,19149 | 0,254489 | 0,216868 | 0,27569 |
| **\|Q03294\|** | 0,073764 | 0,177273 | 0,287421 | 0,405747 | 0,389342 | 1 | 0,795195 | 0,931865 | 0,386483 | 0,337673 | 0,500541 | 0,671748 | 0,372353 | 0,422324 | 0,62237 | 0,368004 | 0,327455 | 0,40202 | 0,369457 | 0,569086 |
| **\|Q03295\|** | 0,084614 | 0,17625 | 0,282418 | 0,412603 | 0,395559 | 0,795195 | 1 | 0,76523 | 0,400357 | 0,314421 | 0,500463 | 0,714183 | 0,386182 | 0,440935 | 0,569077 | 0,361054 | 0,321477 | 0,3943 | 0,362456 | 0,554524 |
| **\|Q04535\|** | 0,072535 | 0,185204 | 0,294039 | 0,391309 | 0,374924 | 0,931865 | 0,76523 | 1 | 0,372139 | 0,342466 | 0,484316 | 0,645868 | 0,364038 | 0,406989 | 0,605361 | 0,373699 | 0,333604 | 0,38742 | 0,375114 | 0,53019 |
| **\|Q26289\|** | 0,046407 | 0,164563 | 0,162947 | 0,619976 | 0,721874 | 0,386483 | 0,400357 | 0,372139 | 1 | 0,222623 | 0,485822 | 0,467975 | 0,572984 | 0,61862 | 0,264324 | 0,227729 | 0,189501 | 0,251867 | 0,214613 | 0,275192 |
| **\|Q25206\|** | 0,052883 | 0,191983 | 0,324407 | 0,191023 | 0,22529 | 0,337673 | 0,314421 | 0,342466 | 0,222623 | 1 | 0,343666 | 0,343196 | 0,181425 | 0,287476 | 0,547372 | 0,447046 | 0,470009 | 0,485581 | 0,478349 | 0,563691 |
| **\|Q03296\|** | 0,06114 | 0,169444 | 0,23084 | 0,505089 | 0,50495 | 0,500541 | 0,500463 | 0,484316 | 0,485822 | 0,343666 | 1 | 0,526318 | 0,480943 | 0,652177 | 0,358989 | 0,299786 | 0,266693 | 0,327838 | 0,297597 | 0,370386 |
| **\|Q04536\|** | 0,064466 | 0,17459 | 0,257861 | 0,486054 | 0,471673 | 0,671748 | 0,714183 | 0,645868 | 0,467975 | 0,343196 | 0,526318 | 1 | 0,46125 | 0,538684 | 0,494753 | 0,355927 | 0,304752 | 0,389847 | 0,351594 | 0,537993 |
| **\|Q04537\|** | 0,038712 | 0,161824 | 0,160562 | 0,637835 | 0,550464 | 0,372353 | 0,386182 | 0,364038 | 0,572984 | 0,181425 | 0,480943 | 0,46125 | 1 | 0,556306 | 0,2573 | 0,224455 | 0,188447 | 0,242783 | 0,213744 | 0,256885 |
| **\|Q26287\|** | 0,054669 | 0,168919 | 0,185396 | 0,586558 | 0,603419 | 0,422324 | 0,440935 | 0,406989 | 0,61862 | 0,287476 | 0,652177 | 0,538684 | 0,556306 | 1 | 0,318041 | 0,269396 | 0,223947 | 0,296899 | 0,256009 | 0,319165 |
| **\|Q25109\|** | 0,056255 | 0,163191 | 0,417886 | 0,274496 | 0,267271 | 0,62237 | 0,569077 | 0,605361 | 0,264324 | 0,547372 | 0,358989 | 0,494753 | 0,2573 | 0,318041 | 1 | 0,604517 | 0,567468 | 0,670772 | 0,612511 | 0,830864 |
| **\|Q25221\|** | 0,046709 | 0,164957 | 0,523844 | 0,242531 | 0,230115 | 0,368004 | 0,361054 | 0,373699 | 0,227729 | 0,447046 | 0,299786 | 0,355927 | 0,224455 | 0,269396 | 0,604517 | 1 | 0,714084 | 0,807202 | 0,722107 | 0,604432 |
| **\|Q25435\|** | 0,058641 | 0,188474 | 0,513149 | 0,204288 | 0,19149 | 0,327455 | 0,321477 | 0,333604 | 0,189501 | 0,470009 | 0,266693 | 0,304752 | 0,188447 | 0,223947 | 0,567468 | 0,714084 | 1 | 0,775997 | 0,783535 | 0,569318 |
| **\|Q26231\|** | 0,053762 | 0,165152 | 0,560201 | 0,266014 | 0,254489 | 0,40202 | 0,3943 | 0,38742 | 0,251867 | 0,485581 | 0,327838 | 0,389847 | 0,242783 | 0,296899 | 0,670772 | 0,807202 | 0,775997 | 1 | 0,784035 | 0,657743 |
| **\|Q26612\|** | 0,058568 | 0,164894 | 0,538353 | 0,239285 | 0,216868 | 0,369457 | 0,362456 | 0,375114 | 0,214613 | 0,478349 | 0,297597 | 0,351594 | 0,213744 | 0,256009 | 0,612511 | 0,722107 | 0,783535 | 0,784035 | 1 | 0,603041 |
| **\|Q27135\|** | 0,048837 | 0,185939 | 0,421066 | 0,271905 | 0,27569 | 0,569086 | 0,554524 | 0,53019 | 0,275192 | 0,563691 | 0,370386 | 0,537993 | 0,256885 | 0,319165 | 0,830864 | 0,604432 | 0,569318 | 0,657743 | 0,603041 | 1 |

**Ribulose bisphosphate carboxylase/oxygenase activase, chloroplastic: distance matrix obtained with *seqpdist***

|  | **\|Q7X9A0\|** | **\|Q40460\|** | **\|Q7X999\|** | **\|Q40565\|** | **\|Q40073\|** | **\|Q42450\|** | **\|P85086\|** | **\|P10896\|** | **\|P23489\|** | **\|Q01587\|** | **\|Q9ZT00\|** | **\|Q40281\|** | **\|P93431\|** | **\|O64981\|** | **\|P84562\|** | **\|O49074\|** | **\|P10871\|** | **\|O98997\|** | **\|P85111\|** | **\|Q7M3P1\|** |
| --- | --- | --- | --- | --- | --- | --- | --- | --- | --- | --- | --- | --- | --- | --- | --- | --- | --- | --- | --- | --- |
| **\|Q7X9A0\|** | 1 | 0,781751 | 0,930039 | 0,768783 | 0,787077 | 0,763956 | 0,183313 | 0,828431 | 0,578409 | 0,762126 | 0,75327 | 0,827409 | 0,813743 | 0,795156 | 0,280627 | 0,753096 | 0,788646 | 0,8115 | 0,208942 | 0,376656 |
| **\|Q40460\|** | 0,781751 | 1 | 0,831792 | 0,952431 | 0,737465 | 0,778507 | 0,161221 | 0,771678 | 0,605845 | 0,781292 | 0,754794 | 0,829055 | 0,754889 | 0,800565 | 0,262595 | 0,874611 | 0,739992 | 0,814309 | 0,211128 | 0,367782 |
| **\|Q7X999\|** | 0,930039 | 0,831792 | 1 | 0,818407 | 0,761333 | 0,799772 | 0,187665 | 0,805309 | 0,609419 | 0,808845 | 0,796044 | 0,865188 | 0,784485 | 0,84262 | 0,25793 | 0,803794 | 0,765812 | 0,861752 | 0,211491 | 0,367873 |
| **\|Q40565\|** | 0,768783 | 0,952431 | 0,818407 | 1 | 0,739406 | 0,779025 | 0,158065 | 0,764869 | 0,598692 | 0,765708 | 0,750029 | 0,812148 | 0,750614 | 0,78882 | 0,275031 | 0,887588 | 0,734327 | 0,808105 | 0,21136 | 0,377272 |
| **\|Q40073\|** | 0,787077 | 0,737465 | 0,761333 | 0,739406 | 1 | 0,821362 | 0,175441 | 0,780421 | 0,586205 | 0,706403 | 0,811047 | 0,752254 | 0,906803 | 0,722056 | 0,273755 | 0,725055 | 0,761724 | 0,734299 | 0,20957 | 0,376816 |
| **\|Q42450\|** | 0,763956 | 0,778507 | 0,799772 | 0,779025 | 0,821362 | 1 | 0,187012 | 0,753209 | 0,613832 | 0,753813 | 0,830892 | 0,793723 | 0,837796 | 0,773097 | 0,282818 | 0,746701 | 0,70277 | 0,777802 | 0,194384 | 0,377418 |
| **\|P85086\|** | 0,183313 | 0,161221 | 0,187665 | 0,158065 | 0,175441 | 0,187012 | 1 | 0,176846 | 0,118936 | 0,161129 | 0,173289 | 0,165428 | 0,18058 | 0,169955 | 0,323147 | 0,145608 | 0,137292 | 0,180709 | 0,46936 | 0,391445 |
| **\|P10896\|** | 0,828431 | 0,771678 | 0,805309 | 0,764869 | 0,780421 | 0,753209 | 0,176846 | 1 | 0,586696 | 0,758534 | 0,730221 | 0,799083 | 0,802494 | 0,765531 | 0,266144 | 0,748658 | 0,778563 | 0,768881 | 0,211834 | 0,367296 |
| **\|P23489\|** | 0,578409 | 0,605845 | 0,609419 | 0,598692 | 0,586205 | 0,613832 | 0,118936 | 0,586696 | 1 | 0,612436 | 0,600522 | 0,615322 | 0,589719 | 0,609785 | 0,283869 | 0,586136 | 0,550828 | 0,614391 | 0,188905 | 0,377783 |
| **\|Q01587\|** | 0,762126 | 0,781292 | 0,808845 | 0,765708 | 0,706403 | 0,753813 | 0,161129 | 0,758534 | 0,612436 | 1 | 0,741055 | 0,819773 | 0,729252 | 0,794502 | 0,283596 | 0,753063 | 0,69844 | 0,796777 | 0,212995 | 0,377688 |
| **\|Q9ZT00\|** | 0,75327 | 0,754794 | 0,796044 | 0,750029 | 0,811047 | 0,830892 | 0,173289 | 0,730221 | 0,600522 | 0,741055 | 1 | 0,779912 | 0,833777 | 0,784058 | 0,282648 | 0,729151 | 0,688647 | 0,780089 | 0,202262 | 0,377358 |
| **\|Q40281\|** | 0,827409 | 0,829055 | 0,865188 | 0,812148 | 0,752254 | 0,793723 | 0,165428 | 0,799083 | 0,615322 | 0,819773 | 0,779912 | 1 | 0,773839 | 0,82636 | 0,282578 | 0,790521 | 0,761805 | 0,842345 | 0,188693 | 0,377334 |
| **\|P93431\|** | 0,813743 | 0,754889 | 0,784485 | 0,750614 | 0,906803 | 0,837796 | 0,18058 | 0,802494 | 0,589719 | 0,729252 | 0,833777 | 0,773839 | 1 | 0,750614 | 0,266271 | 0,736334 | 0,747565 | 0,757913 | 0,209352 | 0,386179 |
| **\|O64981\|** | 0,795156 | 0,800565 | 0,84262 | 0,78882 | 0,722056 | 0,773097 | 0,169955 | 0,765531 | 0,609785 | 0,794502 | 0,784058 | 0,82636 | 0,750614 | 1 | 0,245564 | 0,76079 | 0,736652 | 0,91816 | 0,21136 | 0,282954 |
| **\|P84562\|** | 0,280627 | 0,262595 | 0,25793 | 0,275031 | 0,273755 | 0,282818 | 0,323147 | 0,266144 | 0,283869 | 0,283596 | 0,282648 | 0,282578 | 0,266271 | 0,245564 | 1 | 0,267007 | 0,268697 | 0,282515 | 0,069136 | 0,461695 |
| **\|O49074\|** | 0,753096 | 0,874611 | 0,803794 | 0,887588 | 0,725055 | 0,746701 | 0,145608 | 0,748658 | 0,586136 | 0,753063 | 0,729151 | 0,790521 | 0,736334 | 0,76079 | 0,267007 | 1 | 0,71838 | 0,778717 | 0,210413 | 0,386456 |
| **\|P10871\|** | 0,788646 | 0,739992 | 0,765812 | 0,734327 | 0,761724 | 0,70277 | 0,137292 | 0,778563 | 0,550828 | 0,69844 | 0,688647 | 0,761805 | 0,747565 | 0,736652 | 0,268697 | 0,71838 | 1 | 0,752568 | 0,203995 | 0,423848 |
| **\|O98997\|** | 0,8115 | 0,814309 | 0,861752 | 0,808105 | 0,734299 | 0,777802 | 0,180709 | 0,768881 | 0,614391 | 0,796777 | 0,780089 | 0,842345 | 0,757913 | 0,91816 | 0,282515 | 0,778717 | 0,752568 | 1 | 0,21152 | 0,377312 |
| **\|P85111\|** | 0,208942 | 0,211128 | 0,211491 | 0,21136 | 0,20957 | 0,194384 | 0,46936 | 0,211834 | 0,188905 | 0,212995 | 0,202262 | 0,188693 | 0,209352 | 0,21136 | 0,069136 | 0,210413 | 0,203995 | 0,21152 | 1 | 0,125196 |
| **\|Q7M3P1\|** | 0,376656 | 0,367782 | 0,367873 | 0,377272 | 0,376816 | 0,377418 | 0,391445 | 0,367296 | 0,377783 | 0,377688 | 0,377358 | 0,377334 | 0,386179 | 0,282954 | 0,461695 | 0,386456 | 0,423848 | 0,377312 | 0,125196 | 1 |
